# Growing Pains and Dietary Habits in Young Athletes: A Cross-Sectional Survey

**DOI:** 10.3390/nu17142384

**Published:** 2025-07-21

**Authors:** Carlos Elvira-Aranda, José Antonio Pérez-Turpin, Concepción Suárez-Llorca, Maite Pérez, Roser De-Castellar

**Affiliations:** 1 Research Group on Physical Activity and Sports Sciences, Faculty of Education, University of Alicante, 03690 Alicante, Spain; cea.elvi@gmail.com (C.E.-A.); jose.perez@gcloud.ua.es (J.A.P.-T.); concepcion.suarez@ua.es (C.S.-L.); 2 Kinetic Performance S.L., Scientific Park of Alicante, 03540 Alicante, Spain; 3 Medical Affairs, Laboratorios Ordesa S.L., 08038 Barcelona, Spain; maite.perez@ordesalab.com

**Keywords:** growing pains, musculoskeletal pain, dietary habits, Mediterranean diet

## Abstract

**Background/Objectives**: Growing pains are a common cause of recurrent limb pain in children, but their etiology remains unclear. Physical activity and nutrition are important factors for musculoskeletal health, but their specific relationship with growing pains has not been well established in young athletes. This study aimed to assess the prevalence of growing pains in child and adolescent athletes and evaluate their adherence to the Mediterranean Diet. **Methods**: A cross-sectional study was conducted with 916 athletes aged 8–17 years from sports academies in Alicante, Spain. Data were collected via an online survey assessing demographics, pain types, and adherence to the Mediterranean diet. **Results:** Self-reported pain was highly prevalent, affecting 78.5% of children and 93.5% of adolescents. Musculoskeletal and nocturnal pain increased with age, with nocturnal pain significantly more frequent in girls among children (*p* < 0.001). Additionally, 32.6% of children and 51.9% of adolescents had received a formal diagnosis of growing pains. Despite this, only 13.7% reported using analgesics, with no significant gender differences. Adherence to the Mediterranean diet was mostly moderate, with children reporting higher fruit and vegetable intake than adolescents, while adolescents consumed more healthy fats and carbohydrates, and participants without pain showed overall healthier dietary patterns. **Conclusions**: Idiopathic musculoskeletal pain is highly prevalent among young athletes, and their adherence to a healthy diet is suboptimal, challenging the assumption that physically active children maintain well-balanced diets. This underscores the importance of early nutritional education as a strategy to support musculoskeletal health and reduce pain in physically active youth.

## 1. Introduction

Growing pains are among the most common causes of recurrent limb pain in children, first described by Duchamp in 1823 as “nonspecific musculoskeletal discomfort experienced by children aged 3 to 12 years, with a reported prevalence of 37%” [1], and later defined by Øster and Nielsen (1972) as “intermittent and frequent pain affecting the arms or legs, but not the joints, and disappearing by morning” [2]. Onset typically occurs between the ages of 4 and 14 [3], with reported prevalence ranging from 2.6% to 49.4%, depending on the diagnostic criteria applied [4,5,6].

Despite extensive research, the etiology remains unclear, with multiple theories proposed [7,8]. The growth theory proposes that bone growth triggers musculoskeletal impulses, which become more noticeable at night due to reduced external stimuli. Additionally, the nocturnal peak in growth hormone secretion may contribute to pain perception [9]. The fatigue theory, originally proposed by Bennie in 1894, attributes growing pains to muscle overuse and the accumulation of metabolic byproducts, particularly in active children [10]. The anatomical theory links growing pains to structural abnormalities like scoliosis or flat feet, though evidence is inconclusive [11,12]. The emotional theory suggests a psychosomatic origin, linking growing pains to stress and family dynamics, with affected children often showing higher anxiety levels [13]. Other hypotheses include vitamin D deficiency, joint hypermobility, and a lower pain threshold, but no single cause has been definitively established [14,15,16].

Biological maturation during childhood and adolescence follows distinct sex-specific patterns. Peak Height Velocity (PHV), the period of maximum linear growth, typically occurs earlier in girls (around 11–12 years) than in boys (around 13–14 years), with a lower average growth velocity observed in girls compared to boys, and undergoes a phase of rapid but uncoordinated growth, with lower limb development peaking before that of the torso [17,18]. These maturational differences are well documented and may influence musculoskeletal development during growth [19].

Moreover, during growth, children and adolescents experience physiological stress, which directly affects their developing musculoskeletal system [20,21]. Regular physical activity during this period helps enhance bone mass, muscle strength, and overall skeletal development [22,23]. However, when physical activity becomes particularly intense, it may increase the load on muscles, tendons, bones, and joints that are still maturing. This, combined with the natural physiological stress of growth, may contribute to growing pains [24,25].

On the other hand, adequate nutrition is crucial during growth stages. A well-balanced diet supports optimal growth, tissue repair, and the prevention of deficiency-related conditions such as scurvy, rickets, and malnutrition [26,27], whereas malnutrition and obesity are associated with an increased risk of metabolic and long-term health complications [28,29].

In young athletes, nutritional support becomes even more relevant, as it must sustain both training demands and physiological development [30]. A balanced diet should include carbohydrates for energy, proteins for muscle repair, and healthy fats for endurance, prioritizing nutrient-dense sources such as whole grains, legumes, fish, dairy, and omega-3-rich foods [30,31]. In this regard, the Mediterranean diet has gained attention in sports nutrition due to its anti-inflammatory properties, cardiovascular benefits, and balanced macronutrient profile, potentially aiding in muscle recovery and exercise adaptation [32].

The Mediterranean diet is characterized by a high intake of plant-based foods, moderate consumption of fish and dairy products, and limited intake of processed items. This dietary pattern provides several nutrients involved in bone and musculoskeletal development during growth, including calcium, vitamin D, high-quality proteins, and phosphorus [33,34].

Beyond their individual effects, growing interest exists in the interaction between physical activity and dietary behaviors, and their combined influence on health outcomes such as body composition and musculoskeletal integrity [35]. Adolescence, marked by a mismatch between physical and psychological maturity, is a period especially vulnerable to poor lifestyle choices. Studies in youth have identified coexisting patterns such as the [35,36,37] ”Prudent–Active” and “Western–Sedentary” profiles [35,36,37], highlighting that more active individuals also tend to adopt healthier eating habits. While early established behaviors often persist into adulthood, they remain modifiable [38], underscoring the importance of timely lifestyle interventions [35,37].

Within this context and considering the limited evidence on these topics, particularly in physically active populations, the aim of this exploratory study was to assess the prevalence of growing pains in child and adolescent athletes and, in parallel, describe their adherence to the Mediterranean Diet.

## 2. Materials and Methods

### 2.1. Study Design

An observational, cross-sectional epidemiological study was conducted in male and female athletes aged between 8 and 17 years, in which data were collected through a survey. The study was conducted in five clubs/sports academies from the province of Alicante (Spain), with the participation of the tech-based company Kinetic Performance from Universidad de Alicante in 2022. The inclusion criteria required participants to be competitive-level athletes actively training in a sports club or academy.

The recruitment process involved an informative session with parents, coaches, and team supervisors, during which the objectives of the study were explained, and participation was offered. All athletes who voluntarily agreed to participate completed the questionnaire independently, with parental or guardian assistance when needed. The study was offered to all eligible individuals in the participating academies, and there were no exclusion criteria. The sports practiced by the participants included football, swimming, handball, and volleyball.

Participants completed the online questionnaire independently, with parental or guardian supervision and assistance when needed. All eligible participants who voluntarily responded to the survey were included in the analysis.

The survey consisted of three main sections: one collecting demographic data, including age, gender, and hours of sports practice; another assessing the presence and characteristics of self-reported musculoskeletal pain with no apparent cause; and a third evaluating adherence to the Mediterranean diet using the KIDMED test (Mediterranean Diet Quality Index for children and teenagers) [39].

### 2.2. Pain Questionnaire and Stratification

The pain questionnaire included three close-ended (yes/no) questions designed to identify different aspects of growing pains, based on two of the most widely accepted definitions in the literature. Using Duchamp’s classical description [1], participants were asked: “Normally, do you feel pain or discomfort in muscles, joints, bones, or tendons, even if you haven’t had any injury or fall during sports?”

Based on the description proposed by Øster and Nielsen (1972) [2], nocturnal pain without an apparent cause was recorded. Participants were asked: “Do you suffer from pain in your arms, legs, or back without a known cause, especially at night?”

Finally, to assess clinically diagnosed growing pains, participants were asked: “Has a pediatrician or doctor ever told you that you suffer from growing pains?”

Pain reports were analyzed based on participants’ responses to three specific questions. Whether participants responded affirmatively to at least one of the three questions was also recorded in a combined variable.

Lastly, the questionnaire included an additional question about the use of analgesics or anti-inflammatory drugs for pain relief. Participants were also asked whether they habitually used nutritional supplements.

### 2.3. KIDMED Test

The KIDMED test is a 16-item questionnaire designed to assess adherence to the Mediterranean diet [30]. Each item requires a yes/no response. Affirmative responses to questions reflecting negative dietary habits in relation to the Mediterranean diet received a score of −1 point, while affirmative responses to questions representing positive dietary aspects were scored + 1 point. Negative responses did not contribute to the total score. The final score ranged from 0 to 12, with higher values indicating greater adherence to the Mediterranean dietary pattern.

Based on the total score, participants were classified into three adherence categories: scores above 8 indicated high adherence (reflecting an optimal diet); scores between 4 and 7 indicated moderate adherence (requiring dietary improvement); and scores below 4 indicated low adherence and a poor-quality diet.

For further analysis, the authors grouped individual items into four broader categories to qualitatively assess dietary quality beyond the total score. Although this categorization was not part of the original questionnaire structure, it was developed to explore specific aspects of habitual dietary intake. The categories were defined as follows: fruit and vegetable consumption, indicated by correct responses to items 1, 2, 3, and 4; regular intake of healthy fats, identified by at least two correct responses among items 5, 10, and 11; regular intake of healthy carbohydrates, defined by correct responses to both items 8 and 9; and severe unhealthy habits, characterized by incorrect responses to at least three of the following four items: 6, 12, 14, and 16.

### 2.4. Endpoints and Variables

The primary endpoints of the study were the prevalence of idiopathic musculoskeletal pain in young athletes, categorized as present or absent.

Secondary endpoints included the qualitative assessment of adherence to the Mediterranean diet, assessed through the KIDMED test, as well as the evaluation of the relationship between pain and dietary adherence as potential risk factors.

### 2.5. Statistical Analysis

The results were stratified by age (children: 8–12 years; adolescents: 13–17 years old) and gender (male or female). Comparisons involving continuous variables, such as weekly hours of sports practice and analgesic use, were assessed using the t-test or Mann–Whitney U test, depending on normality (Shapiro–Wilk test). Associations between categorical variables related to the presence of pain were analyzed using the Chi-square test. A *p*-value of < 0.05 was considered statistically significant. Figures on general dietary adherence and eating habits (KIDMED index) were presented descriptively. Statistical analysis was only applied when these variables were compared by pain status.

## 3. Results

### 3.1. Characteristics of the Participants

A total of 916 participants, aged between 8 and 17 years, were included in the study. The distribution of sports disciplines reflected the recruitment strategy, with the highest number of participants engaged in football (n = 309), owing to the inclusion of two football clubs. This was followed by handball (n = 258), volleyball (n = 196), and swimming (n = 153). Football was the most frequently practiced discipline across both age groups, while handball was more common among children, and volleyball among adolescents. Adolescents reported significantly more weekly training hours than children, with nearly double the time dedicated to sports (*p* < 0.001). Additionally, 94.3% of participants (n = 864) reported not using nutritional supplements to recover from training or competitions.

The baseline characteristics of the participants are summarized in Table 1.

Regarding height, females presented greater average height values between the ages of 10 and 14, while males presented higher values from age 15 onwards (Figure S1).

### 3.2. Prevalence of Pain Based on Questionnaire Responses

Notably, a relevant proportion of participants reported experiencing pain, with 820 out of 916 individuals affected. The overall prevalence was significantly higher in adolescents compared to children (93.5% vs. 78.5%; *p* < 0.001). Despite this, the use of analgesics was relatively limited, with 13.7% of participants reporting the use of palliative treatments. Analgesic use followed a similar pattern to pain prevalence, with higher rates observed in adolescents (15.1% vs. 9.5%; *p* < 0.05) (Table 1).

**Figure 1:**
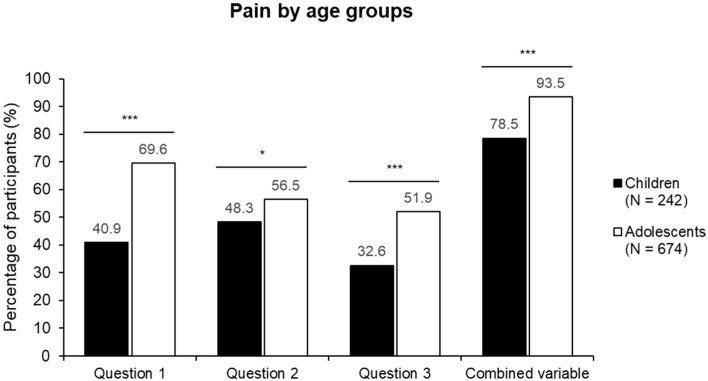
Distribution of participants reporting pain by age group. Question 1 refers to musculoskeletal pain without an apparent cause, assessed by asking, “Do you feel pain or discomfort in muscles, joints, bones, or tendons, even if you haven’t had any injury or fall during sports?” Question 2 refers to nocturnal pain without an apparent cause, assessed by asking, “Do you suffer from pain in your arms, legs, or back without a known cause, especially at night?”, and Question 3 refers to diagnosed growing pains, “Has a pediatrician or doctor ever told you that you suffer from growing pains?”. The combined variable includes participants who responded affirmatively to at least one of the three questions. * *p* < 0.05, *** *p* <0.001. All comparisons were conducted using the Chi-square test.

The proportion of participants reporting pain was significantly higher in the adolescent group than in the younger one across all pain-related questions (Figure 1).

In relation to Question 1, which refers to musculoskeletal pain with no apparent cause, no gender differences were observed in the 8–12 years group. However, among adolescents, the prevalence was significantly higher in males than in females (73.6% vs. 65.5%; *p* = 0.042).

Regarding nocturnal pain (Question 2), a significant gender difference was observed only in the younger group, where females reported symptoms much more frequently than males (64.9% vs. 33.6%; *p* < 0.001). No significant differences were found between genders in adolescents.

As for Question 3, which addressed whether participants had ever been diagnosed with growing pains, no significant differences between males and females were identified in either age group. Similarly, no gender-related differences were found in the combined variable, which included participants who responded affirmatively to at least one of the three questions.

**Figure 2:**
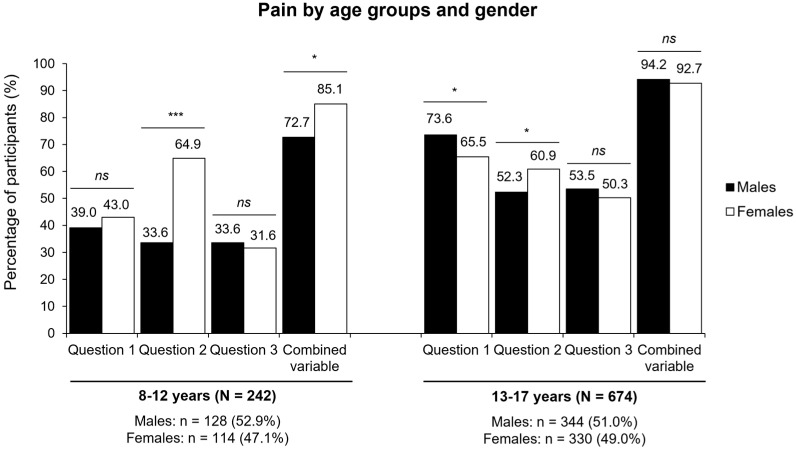
Percentage of participants experiencing pain. Distribution of participants reporting pain classified by age group and gender. Question 1 refers to musculoskeletal pain without an apparent cause, assessed by asking, “Do you feel pain or discomfort in muscles, joints, bones, or tendons, even if you haven’t had any injury or fall during sports?” Question 2 refers to nocturnal pain without an apparent cause, assessed by asking, “Do you suffer from pain in your arms, legs, or back without a known cause, especially at night?”, and Question 3 refers to diagnosed growing pains, “Has a pediatrician or doctor ever told you that you suffer from growing pains?”. The combined variable includes participants who responded affirmatively to at least one of the three questions. * *p* < 0.05; *** *p* < 0.001; *ns*, non-significant. All comparisons were conducted using the Chi-square test.

Figure 2 illustrates the distribution of pain by age groups and gender. Overall pain prevalence by gender and age and the distribution of the responses to the different questions divided by year are summarized in Figures S2 and S3. Additionally, analgesic use differed significantly between children and adolescents, with older participants reporting higher use (*p* = 0.029). In contrast, no significant differences in analgesic use were observed between sexes within each age group (Supplementary Table S1).

### 3.3. Dietary Habits

**Figure 3:**
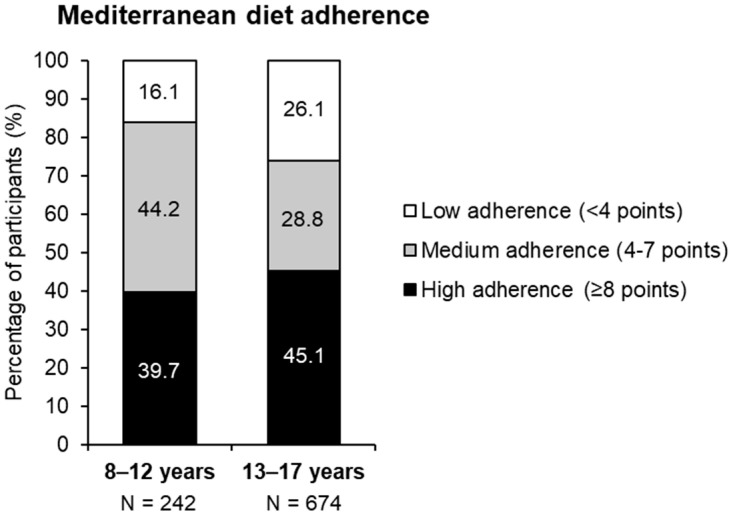
Adherence to Mediterranean diet by age. Distribution of participants according to different adherence levels. Adherence was classified based on the KIDMED test score as high (≥8 points), medium (4–7 points), or low (<4 points). Data are presented descriptively without statistical significance analysis.

High and medium adherence to the Mediterranean diet were mostly reported by both children and adolescents, as shown in Figure 3.

To further analyze dietary habits, individual responses were categorized into broader food consumption patterns.

**Figure 4:**
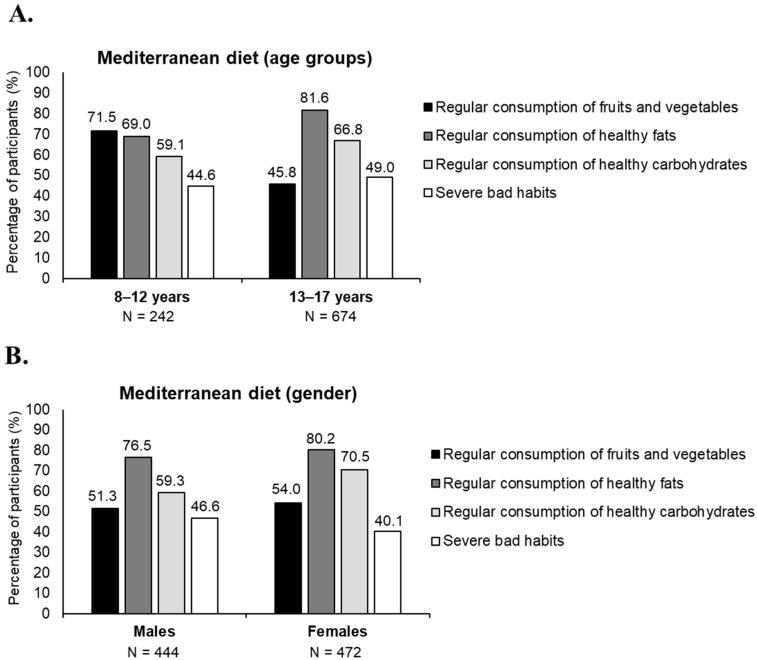
Dietary habit categories by age group and gender. Percentage of participants classified into different dietary habit categories based on their responses to the KIDMED questionnaire, stratified by age groups (**A**) and gender (**B**). Participants could be included in multiple categories based on their dietary responses. Data are presented descriptively without statistical significance analysis.

Dietary patterns varied across age and gender groups. Up to 71.5% of children reported regular intake of fruits and vegetables, while the figure was 45.8% in adolescents. However, the regular consumption of healthy fats was 69.0% for the younger group and 81.6% for adolescents (Figure 4A). The same descriptive analysis was performed based on gender, as shown in Figure 4B.

**Figure 5:**
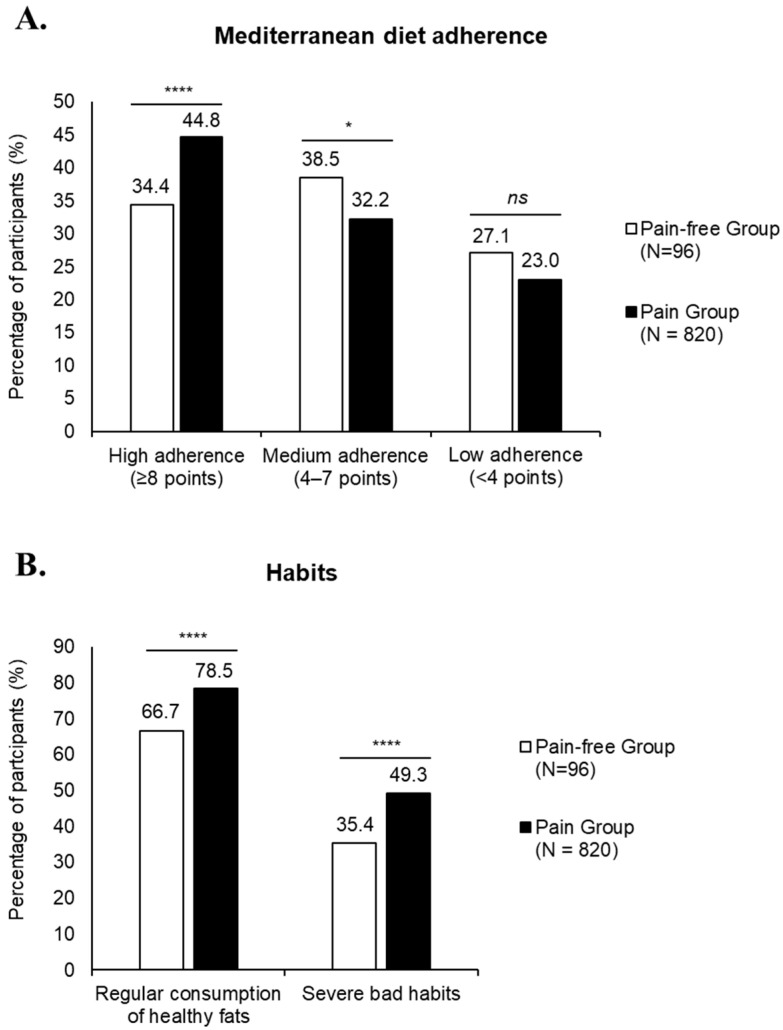
Mediterranean diet adherence and dietary habit categories and presence of pain. (**A**) Distribution of participants by adherence to Mediterranean diet, classified as high (≥8 points), medium (4–7 points), or low (<4 points) (**A**), and percentage of participants classified into different dietary habit categories (**B**). Data are classified based on KIDMED test and stratified according to whether participants reported pain or not. Participants could be included in multiple categories based on their dietary responses. * *p* < 0.05; **** *p* < 0.0001; *ns*, non-significant. All comparisons were conducted using the Chi-square test.

A comparison of Mediterranean diet adherence and dietary habits between participants with and without pain revealed significant differences. Participants with pain showed higher adherence to the Mediterranean diet; however, unhealthy dietary habits were also more frequent in this group (Figure 5).

## 4. Discussion

Nutrition and physical activity are well-established factors influencing growth and athletic performance. At the same time, musculoskeletal stress and growth-related changes might contribute to musculoskeletal discomfort in young athletes. This exploratory study aimed to assess the prevalence of growing pains in child and adolescent athletes and, in parallel, describe their adherence to the Mediterranean diet.

A total of 916 participants completed the survey, exceeding common methodological recommendations for exploratory observational studies [40], and the size and balance of the sample across age and sex groups were adequate for the scope of the analyses conducted.

The results of this study indicate a notably high prevalence of idiopathic musculoskeletal pain in children and adolescents engaged in regular sports practice—exceeding 70% in children and 90% in adolescents. These values exceed those reported in the general pediatric population (11–15%, occasionally 33–44%) [6,41], likely reflecting the added physical demands of competitive sports, even at amateur levels. This highlights the need to address the specific physiological challenges faced by young athletes.

Moreover, the proportion of participants reporting musculoskeletal pain (Question 1) was significantly higher in adolescents, coinciding with a greater number of weekly training hours observed. This difference suggests that higher training loads and physical demands during adolescence may contribute to pain development. Similar observations have been made in previous studies involving both adolescents [42] and physically active children [43], but the prevalence reported here remains remarkably high. This may be influenced by factors such as training volume and intensity, sport-specific biomechanical demands, or inadequate recovery periods, all of which warrant further investigation.

The gender difference observed in nocturnal pain prevalence (Question 2) was more pronounced in children, with females reporting symptoms nearly twice as often as males. Since pubertal onset occurs earlier in females than males (10.5–11 vs. 12–13 years) [17], this disparity could be partly attributed to hormonal factors. Initial increases in estrogen in females might contribute to heightened pain sensitivity by modulating nociceptive pathways, whereas boys experience a later rise in testosterone, which has been associated with analgesic effects [44]. During adolescence, as musculoskeletal maturation progresses and hormonal influences become more balanced, these differences in pain perception may diminish.

Additionally, in our study, females showed numerically greater average height values between the ages of 10 and 14, which aligns with the expected timing of earlier biological maturation. During the adolescent growth spurt, PHV in females may lead to temporary musculoskeletal imbalances and increased pain perception [19]. In a study in children and adolescents aged 10 to 15 years, girls also reported higher pain intensity than boys, a difference partially explained by psychosocial factors [45]. However, due to the descriptive and cross-sectional nature of our study, these findings should be interpreted with caution, as no causal inferences can be made regarding the origin of these differences, and further research is warranted.

Another relevant observation is the high proportion of participants with a well-established clinical diagnostic of growing pains (Question 3), affecting 30% of children and 50% of adolescents. Although pain susceptibility could influence self-reported data, these results point to a clinically relevant issue that may be underestimated in young athletes. Because this prevalence derives from clinical diagnoses, it is more directly comparable to previous studies that assessed growing pain prevalence using medical consultation records [4,5,6]. This contrasts with the findings from Question 1, which reported a prevalence of up to 90% in adolescents. As participants in this study were surveyed in a sport setting rather than a clinical environment, the sample may include children and adolescents who do not typically seek medical care for musculoskeletal complaints. This setting may facilitate the identification of otherwise unrecognized cases, thereby revealing a segment of the population affected by growing pains that remains underdiagnosed within the healthcare system.

Despite the frequency of reported pain, most participants did not take any measures to alleviate their symptoms. These findings align with recent studies indicating that pain management generally relies on non-pharmacological approaches, such as massages or stretching exercises [8,46], but contrast with earlier research suggesting that 52% of children should be treated with analgesics [47]. The combination of high pain prevalence and low reliance on analgesics suggests that many families and young athletes perceive pain as a normal consequence of physical activity, often relying instead on passive management strategies [48].

In light of these findings, we assessed adherence to the Mediterranean diet as a global indicator of dietary quality in this population of young athletes. Given its anti-inflammatory properties and potential benefits for musculoskeletal health and performance [32,49], adequate adherence to the Mediterranean diet could help mitigate some of the physical stressors associated with growing pains, particularly in young athletes undergoing high training loads.

Our results suggest that children tend to exhibit a higher overall adherence to the Mediterranean diet. These findings are consistent with a study conducted in Spain, which observed moderate adherence among primary school students, with even higher values in children who practiced sports [50]. Similarly, a study from northern Spain also found greater adherence among younger adolescents. However, their results differ from ours in that, in our population, high adherence increases with age, and the combined proportion of low and medium adherence is somewhat lower [51]. Our results also contrast with research conducted across Europe, which reported higher adherence among adolescents compared to younger children [52].

Interestingly, participants with musculoskeletal pain demonstrated higher adherence to the Mediterranean diet. Most studies have reported an association between lower adherence to the Mediterranean diet and higher levels of pain, but these were conducted in adults [53,54,55], and to our knowledge, no studies to date have examined this relationship in pediatric populations [34]. Further research is needed to explore this association in younger age groups.

Simultaneously, unhealthy dietary habits, such as fast-food consumption, skipping breakfast, and frequent intake of industrial pastries or sweets, were also particularly prevalent among participants reporting pain. These patterns are known to increase the risk of coronary heart disease and cardiovascular events [56], and their occurrence in a physically active cohort challenges the assumption that sports participation guarantees healthy eating [57]. Such nutritional imbalances may impair recovery, promote inflammation, and intensify pain perception, particularly in adolescents with high training loads.

Although causality cannot be established, these findings suggest a potential link between dietary quality and pain, underscoring the complexity of dietary patterns in young athletes, and highlight the need for targeted studies exploring whether optimizing nutritional intake improves adaptation to training stress and reduces musculoskeletal discomfort. Further studies, ideally with longitudinal designs and multivariate analytical approaches, are needed to clarify whether optimizing nutritional intake improves adaptation to training stress and reduces musculoskeletal discomfort.

In parallel, reinforcing nutritional education is crucial, particularly through school-based interventions that promote healthy eating habits. Evidence suggests that structured educational strategies can effectively improve children’s dietary behaviors and knowledge, leading to long-term health benefits [58,59]. In this context, emphasizing the Mediterranean diet and its anti-inflammatory properties may play a key role in optimizing muscle recovery and meeting the nutritional needs of physically active children.

Considering the physiological demands of growth and intense training, future research should explore whether a balanced diet alone is sufficient or if targeted supplementation could provide additional benefits. While a well-balanced diet generally meets young athletes’ needs, certain supplements may provide complementary support. In this regard, studies show that creatine supports explosive performance [60], beta-alanine delays fatigue [61,62], and hydrolyzed collagen aids bone and muscle recovery [63,64,65]. However, these supplements should complement rather than replace a well-balanced diet.

Other studies should also examine how different types of sport affect musculoskeletal pain, distinguishing between endurance disciplines (e.g., running, cycling, swimming) and impact-based explosive sports (e.g., football, basketball, volleyball). This could help identify sport-specific risk patterns and inform more tailored preventive strategies.

A key strength of this study is its focus on a physically active cohort, providing novel insights into the prevalence of musculoskeletal pain in young athletes and its potential association with dietary habits. Unlike most research on the general population, this study specifically examines young athletes, offering new insights into a population often presumed to follow healthier routines due to their level of physical activity. Moreover, the study adopts a broader definition of musculoskeletal pain, extending beyond the narrow clinical interpretation that is sometimes limited to conditions like Osgood–Schlatter disease. Additionally, the analysis stratified by age and gender offers a more detailed understanding of developmental differences, particularly during puberty, when hormonal and musculoskeletal changes may influence pain perception and nutritional needs.

However, some limitations should be considered. The use of self-reported data may introduce recall bias, especially in younger children assisted by parents. Moreover, although participants could self-identify beyond binary gender categories, none did so, which may reflect underrepresentation or reluctance to disclose. In addition, nutrition was assessed using the KIDMED index, without dietary records or clinical data. Therefore, the results reflect general dietary quality, not specific nutrient intake. Lastly, statistical comparisons focused on pain-related variables, which may limit the interpretation of age- or sex-based dietary differences.

## 5. Conclusions

In conclusion, this study highlights the high prevalence of idiopathic musculoskeletal pain in young athletes and underscores the need to address modifiable factors such as nutrition. The persistence of unhealthy dietary habits challenges the common assumption that sports participation alone ensures adequate dietary quality. These findings emphasize the importance of early nutritional education and preventive strategies to promote musculoskeletal health, enhance recovery, and potentially reduce pain incidence in physically active youth.

## Figures and Tables

**Table 1:** Baseline characteristics of participants included in the study.

	8–12 Years (N = 242)	13–17 Years (N = 674)
Males, n (%)	128 (52.9)	344 (51.0)
Females, n (%)	114 (47.1)	330 (49.0)
BMI (kg/m^2^), mean	18.2	20.1
Practiced sport ^a^ (hours), mean ± SD	3.7 ± 1.1	7.2 ± 1.2
Use of nutritional supplements, n (%)	0 (0%)	52 (7.7%)
Participants experiencing pain ^b^, n (%)	190 (78.51)	630 (93.47)
Use of analgesics ^c^, n (%)	23 (9.5)	102 (15.1)

^a^ *p* < 0.001; Mann–Whitney U test. ^b^ Refers to the combined variable, including participants who responded affirmatively to at least one of the three questions in the pain questionnaire (*p* < 0.001; Chi-square test). ^c^ *p* = 0.029; Chi-square test. BMI: Body Mass Index.

## Data Availability

The data presented in this study are available on request from the corresponding author due to privacy concerns.

## Supplementary Materials

The following supporting information can be downloaded at https://www.mdpi.com/article/10.3390/nu17142384/s1, Figure S1: Mean height distribution by age and gender; Figure S2: Distribution of pain questionnaire responses by age; Figure S3: Percentage of participants reporting pain by age and gender; Supplementary Table S1: Use of analgesics by age groups and gender.

## Abbreviations

The following abbreviations are used in this manuscript:

BMI	Body Mass Index
KIDMED	Mediterranean Diet Quality Index for children and teenagers
PHV	Peak Height Velocity
