# Supplementary material for: Growing Pains and Dietary Habits in Young Athletes: A Cross-Sectional Survey
Source: Nutrients. 2025 Jul 21;17(14):2384. doi: 10.3390/nu17142384 (PMC12299381; doi:10.3390/nu17142384)
Supplement: Supplementary file 1 [file nutrients-17-02384-s001.zip › nutrients-3680638-supplementary.pdf]

Supplementary Materials

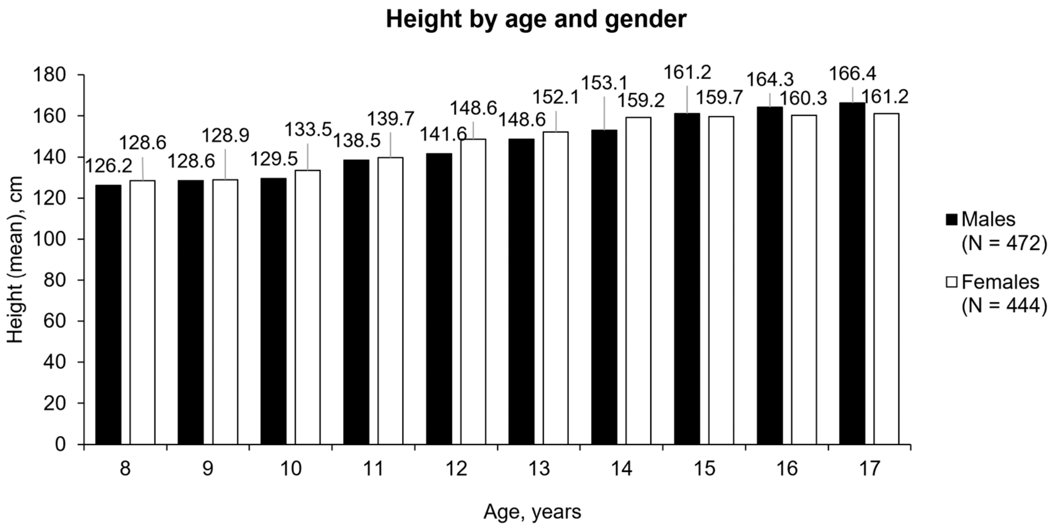

Figure S1. Mean height distribution by age and gender. Distribution of the average height of the participants, shown by individual age (from 8 to 17 years) and stratified by gender. Data are expressed as percentages. Data are presented descriptively without statistical significance analysis.

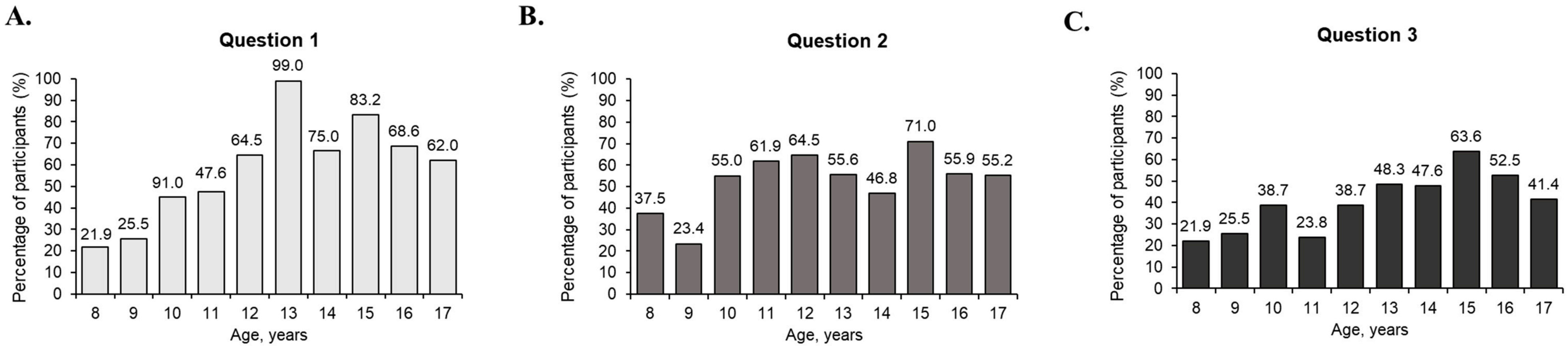

Figure S2. Distribution of pain questionnaire responses by age. Percentage of participants reporting pain by individual age for each questionnaire item. (**A**) Question 1 refers to musculoskeletal pain without an apparent cause, assessed by asking: "*Do you feel pain or discomfort in muscles, joints, bones, or tendons, even if you haven't had any injury or fall during sports?*". (**B**) Question 2 refers to nocturnal pain without an apparent cause, assessed by asking: "*Do you suffer from pain in your arms, legs, or back without a known cause, especially at night?".* (**C**) Question 3 refers to diagnosed growing pains, assessed by asking: "*Has a pediatrician or doctor ever told you that you suffer from growing pains?*". Participants may be included in multiple categories. Data are presented descriptively without statistical significance analysis.

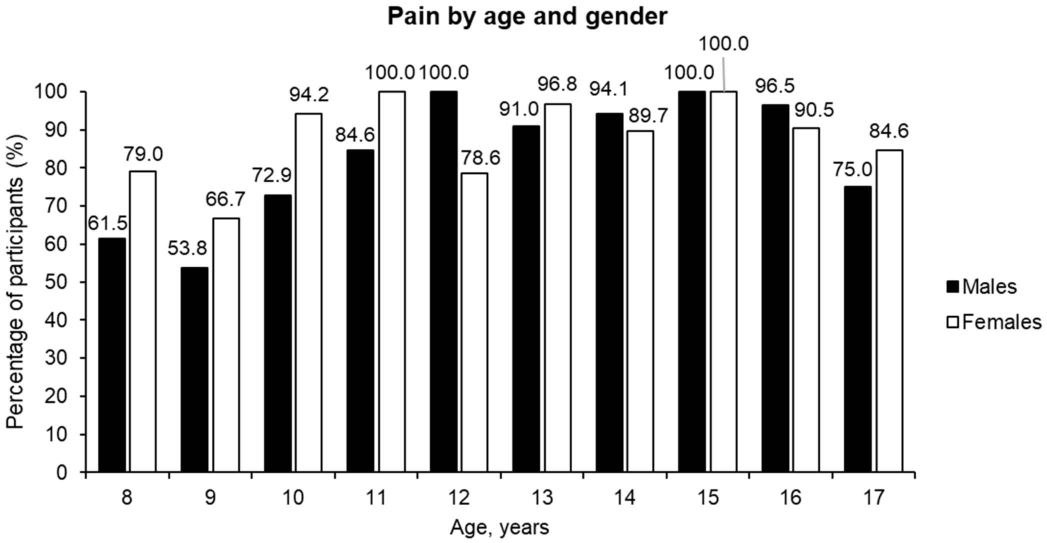

Figure S3. Percentage of participants reporting pain by age and gender. Distribution of participants who responded affirmatively to at least one of the three pain-related questions (combined variable), shown by individual age (from 8 to 17 years) and stratified by gender. Data are expressed as percentages. Data are presented descriptively without statistical significance analysis.

Supplementary Table S1. Use of analgesics by age groups and gender.

Use of analgesics	
**8 – 12 years (N= 242)**	
Males (n = 128), n (%)	10 (7.8)
Females (n = 114), n (%)	13 (11.4)
p-value	0.342
**13 – 17 years (N=674)**	
Males (n = 344), n (%)	55 (16.0)
Females (n = 330), n (%)	47 (14.2)
p-value	0.527

Percentage of participants reporting the use of analgesics or anti-inflammatory drugs, stratified by age group (8–12 years and 13–17 years) and gender. Statistical comparisons between males and females within each age group were conducted using the Chi-square test.
